# Using Public Funeral and Obituary Listings to Identify Spikes in Excess Mortality in One Appalachian County

**DOI:** 10.13023/jah.0603.03

**Published:** 2024-10-01

**Authors:** Allen Archer, Melissa White, Megan Quinn, Randy Wykoff

**Affiliations:** East Tennessee State University; East Tennessee State University; East Tennessee State University

**Keywords:** Appalachia, COVID-19, excess mortality, funeral, obituary, Tennessee

## Abstract

**Introduction:**

Delays (10–22 months) in availability of official state and county-level mortality data could have significant public health consequences. The COVID-19 pandemic illuminated the need for health officials to access timely death data to identify unexpected increases in mortality in their communities.

**Purpose:**

The purpose of this study is to determine if funeral home listings and/or newspaper obituaries could help identify excess mortality on the local level, prior to the availability of official death records.

**Methods:**

To calculate excess mortality, four years (2017–2020) of data were collected from three sources: the state health department, online funeral home listings, and newspaper obituaries, all from Washington County, Tennessee. Simple linear regression was used to predict number of expected deaths by month for 2020 using 2017, 2018, and 2019 reported deaths, by data source. The percent difference of actual 2020 deaths from the expected deaths was then calculated by month and compared for each data source.

**Results:**

Official COVID-19 state-reported death data accounted for only 50% of excess mortality estimated in 2020. Nearly 100 excess deaths occurred before the first reported death due to COVID-19. Trends in the percent difference between actual and expected funeral home listings and newspaper obituaries followed similar patterns as percent differences in actual v. expected state-reported mortality data.

**Implications:**

Had funeral home listings and newspaper obituaries been used to identify excess mortality, health officials would have seen increases in mortality nearly five months prior to the first identified COVID-19 death. These publicly available tools could prove valuable to local health officials as an “early warning” sign of excess mortality.

## INTRODUCTION

In 2020, the Tennessee Department of Health (TDH) reported 6,760 deaths from COVID-19 in the state, the first being reported on March 20.^1^,^2^ Confirmed COVID-19 deaths primarily capture deaths directly resulting from COVID-19, but do not completely capture indirect deaths resulting from the sociocultural impact of the COVID-19 pandemic.^3^ An estimate of excess deaths, defined as the difference between the actual number of deaths in a specific time and the expected number of deaths in the same period, is essential to understand the full impact of the pandemic.^4^ Recognizing excess deaths in a community is important both to assess the overall impact of a disease (pandemic or otherwise) and as an early indicator that a new disease or condition may be impacting a community.

While state-reported mortality data are the gold standard for assessing excess deaths in any area, these data are not readily available during an event associated with a short-term increase in mortality.^5^ In Tennessee, mortality data are officially reported in October for the previous year, meaning that these data are anywhere from 10 to 22 months delayed in their reporting. The availability of mortality data can also vary widely by location and cause of death.^6^

Washington County, Tennessee is located in the Appalachian Region of Northeast Tennessee. 7 As of the 2020 census, the county had a population of just over 130,000 people, with 88% white/non-Hispanic, 4% black/non-Hispanic and 4% Hispanic.^7^ The capital is Jonesborough, although its largest city is Johnson City, which is a part of the Tri-Cities combined statistical area.^7^ Thirty three percent of the population has a postsecondary degree, compared to Tennessee’s overall rate of 29%.^7^ Median household income from 2017–2021 was $52,503, less than the Tennessee rate of $58,507.^7^

It is critical for local public health officials to have access to timely, accurate data to identify and potentially respond to unexpected increases in mortality in their communities. The COVID-19 pandemic provided a unique opportunity in Washington County, Tennessee, to evaluate the utility of online listings of recent deaths by local funeral homes and obituaries published in local newspapers for identifying excess mortality in a timely way.

## METHODS

### Data Sources

Four years of (1) total mortality data for Washington County, Tennessee were formally requested and obtained from TDH; (2) publicly available death listings were collected from four funeral homes in Washington County; and (3) obituary listings were compiled from a local newspaper.^8^ There are a total of 18 funeral homes in Northeast Tennessee. Three of the funeral homes selected were identified as the most utilized in Northeast Tennessee, and the fourth was selected because it was the most utilized by the region’s non-Hispanic black or African American population. Obituary listings were identified from the newspaper with the greatest local circulation, the *Johnson City Press*. Newspaper data were obtained from indices maintained by the Johnson City Public Library and were quality checked by randomly identifying 10% of listings for review on the *Johnson City Press* online archive website.

### Calculating Excess Mortality

Month-by-month deaths for 2017, 2018, 2019, and 2020 were determined for state-level mortality data, online funeral home listings, and newspaper obituaries. There are multiple methodological options for predicting excess mortality to include techniques such as Poisson regression and Bayesian modeling, depending on the type of data and sample size. Due to the small number of data points, limited data, and the aim of this project, simple linear regression models were used to predict excess mortality. Using simple linear regression models, deaths by month for 2017–2019 (as reported by funeral homes and newspaper obituaries) were used to predict expected deaths each month of 2020 for each data source. The slope of the linear regression model was determined, and based on the calculated slope, projected deaths for the corresponding month in 2020 were predicted. This calculation provided the potential number of excess deaths in 2020, which then allowed the calculation of excess deaths. By subtracting expected 2020 monthly deaths from the actual (reported) monthly deaths, monthly number of excess deaths for 2020 were calculated for each data source. This calculated difference was then divided by the expected deaths for the same month to calculate percent difference. This methodology was used to calculate the percent difference in actual and expected deaths for each data source.

*Excess Death Calculation*


(2020 Actual-2020 Expected)/2020 Expected=Difference*100=% Difference

Month-by-month trends in percent differences for TDH mortality data, funeral home death listings, and newspaper obituaries were then graphed to visually represent whether the two alternative mortality data sources had similar peaks and valleys with the official state-level data.

## RESULTS

### Tennessee Department of Health Mortality Data

As shown in Table 1, in 2020, there were a total of 322 excess deaths in Washington County (1,489 expected v. 1,811 actual) representing a percent difference of 21.63% expected v. actual deaths. For the same year, TDH officially reported 165 deaths from COVID-19 in Washington County, with the first occurring on July 28, 2020.^2^ These 165 deaths attributed to COVID-19 represented only 51.24% of the 322 excess deaths in the county in 2020, leaving a remaining 157 or 48.76% of excess deaths not directly attributed to COVID-19.

There was a 7.68% and 18.85% increase in actual v. expected deaths for quarters one and two, respectively. For these two quarters, none of the 96 excess deaths were explained by COVID-19, as no COVID-related deaths had yet been reported in Washington County. Quarters three and four had a 12.21% and 48.91% increase in deaths, respectively. Of the 47 excess deaths in quarter three, 38 (80.28%) were attributed to COVID-19, leaving about 20% unexplained by COVID-19. Similarly, of the 179 deaths in quarter four, 127 (70.82%) were attributed directly to COVID-19, leaving nearly 30% of excess deaths unexplained by COVID-19.

### Funeral Home Listings

As shown in Table 2, there were a total of 911 expected funeral home listings in 2020 compared to 1,037 observed listings, indicating an increase of 126 (13.79%) actual funeral home listings. There was a −9.29%, 11.50%, 7.36% and 55.90% difference in actual v. expected funeral home listings for quarters one, two, three, and four, respectively, in 2020.

### Newspaper obituaries

The expected number of newspaper obituaries for all of 2020 was 1,550 compared to 1,864 actual obituaries, represented by a 20.28% increase (Table 3). By quarter, 2020 actual newspaper obituaries differed from the expected by 8.05%, 20.00%, 9.93% and 44.55% for quarters one, two, three, and four, respectively.

### Comparison of Trends in Mortality between Data Sources

**Figure 1:**
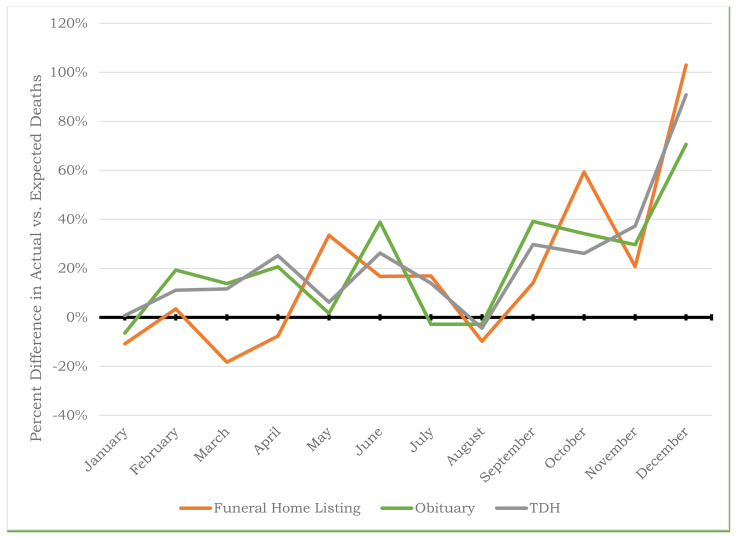
Percentage Difference in Actual v. Expected Monthly Deaths in 2020 by Data Source[Table t4-jah-6-3-4] **Table t4-jah-6-3-4:** Figure 1 Data Table

Figure 1 graphically compares trends in the percent difference in actual v. expected TDH mortality data and the two alternative death data sources: funeral home listings and newspaper obituaries. The trend in percent difference in actual v. expected funeral home listings was consistent with state health department data and the first major spike occurring in May 2020, along with two additional larger spikes in October and December 2020 (Fig. 1). Newspaper obituaries even more closely resembled the TDH data for Washington County (Fig. 1).

## DISCUSSION

This study documents that online public reports from selected funeral homes and newspaper obituary listings are quite accurate in providing local health officers with useful, accessible, and reliable “real time” indications of excess mortality within their region. When visually comparing online funeral listings and newspaper obituaries to state reported data for Washington County, either of the public data sources could have been used to identify spikes in excess mortality throughout 2020. Creating a historical month-by-month registry of the number of deaths in the region, collated from a selected number of funeral homes and/or newspaper obituaries, is an inexpensive and relatively quick method for local health officials to have an early indication that something has changed in their region. Aside from minor differences in February and June, the trends in the percent difference in funeral home listings and official state mortality data in Figure 1 are remarkably similar. Therefore, had this technique for identifying excess mortality been employed prior to the pandemic, health officials in Washington County, Tennessee would have begun seeing increases in mortality as early as February 2020. These trends would have only later, in October 2021, been corroborated by state health department data. Remarkably, almost 100 excess deaths were reported prior to the first identified case of COVID-19 in Washington County.

It was not until August 2020 that the percent of excess deaths attributed to COVID-19 reached the 75–88% predicted by the CDC, indicating considerable under-recognition and significant delays in identifying COVID-19-related deaths in Washington County, Tennessee.^9^ The failure or delay in recognition of excess deaths can have multiple public health implications. For example, in the case of COVID-19, delay in recognizing excess mortality may have contributed to difficulty in convincing the public of the significance of the pandemic, especially in the face of suggestions coming from national-level officials and news outlets that the severity of the pandemic was being exaggerated.^9^,^10^ During non-pandemic times, failure to recognize excess mortality could result in delays in recognizing, or even failure to recognize, an unanticipated cause of death in a community. Because state-level death data can take over a year to be made available to local public health officials, they are not particularly useful for local and state-level health officials in identifying and responding to unexpected changes in the number of deaths in a region.

While this study was conducted during the COVID-19 pandemic, when it was widely recognized that there were going to be excess deaths, the results would be applicable in a wide variety of settings such as the introduction of drugs laced with Fentanyl, an unrecognized contamination of drinking water or other consumer product, or an environmental pollutant. Based on these findings, it appears to be beneficial for health officials to create and maintain a database of deaths at a city or county level, based on funeral home data and newspaper obituaries that could serve as an early indicator that a change has happened in the number of deaths in their region.

### Limitations

Neither funeral home nor obituary data are an accurate measure of actual deaths in a region. Not everyone who dies will have their funeral service in the region where they die and vice versa. Additionally, programs like the Federal Emergency Management Association’s funeral assistance program could have changed the number of deaths reported in funeral home listings, if a funeral home was participating in that program. Obituaries also have a cost associated with them and therefore may be inconsistent depending on other economic priorities. Funeral home and obituary data may also have delays in being published depending on location and other influences, such as the COVID-19 pandemic. Furthermore, it is not possible to identify the cause of excess mortality from these data sources. Finally, while these data sources may be quicker than official data sources, the lag time with these data sources is not fully known and was not determined as part of this project. That said, funeral home and obituary data could be considered tools in understanding trends in mortality and should not be used as the sole source of data to determine mortality rates.

The methodology chosen was used due to the limited data and the aim to provide simple, straightforward estimates for local and regional health departments to determine if excess deaths were occurring during the COVID-19 pandemic. Creating simple linear regression models with only three years of data lends itself to potential inaccuracies and the findings should be interpreted with caution. This was done because, at the time of data collection, online funeral home listings prior to 2017 were unattainable. However, since the method for establishing 2020 monthly expected deaths was replicated for all data sources using the same number of years, it is believed that the comparisons provided are valid. This limitation would also be addressed in time, given that the longer this data is collected, the more years of data would be available for analyses, thereby strengthening confidence in expected death calculations. Further, if additional data were available, other methodologies to predict excess mortality could have been considered, such as Poisson regression, which is typically used for count data. However, the low number of data and the main purpose of the current study limited the applicability of such an approach. It should also be noted that depending on the data and methodology used, excess mortality estimates may vary. For example, a recent study by Paglino et al^11^ provided different excess mortality estimates for Washington County than the current study. These differences are based on the data used in each of the respective studies, the methodologies employed to estimate excess mortality, and the time periods addressed by each study. Methodologies for calculating excess mortality have been a point of discussion for researchers during the COVID-19 pandemic to try to estimate the impact of the pandemic. Vandenbroucke and Pearce recently provided insights into using different methodologies and stated that it is important to identify best practices in calculating excess mortality for different situations.^12^

Finally, while online funeral listings and newspaper obituaries were readily available data sources in Washington County, the utilization and composition of public death listing sources in each region may vary. This study should be considered a pilot study and, should public health professionals seek to employ these techniques for identifying excess mortality, they should identify the most utilized and reliable data sources available in their region.

## CONCLUSION

This study provides preliminary evidence that online funeral home listings and newspaper obituaries provide an accessible and inexpensive “real time” mechanism to identify trends in excess mortality. The technique outlined in this project is one option that could be used in the future to identify future public health threats and ultimately prevent the loss of life at the city/county level, and thus have significant implications for local and state-level public health leaders as well as communities.

**SUMMARY BOX**

**What is already known about this topic?**

Excess mortality is currently calculated a variety of ways, using state or nationally reported death data with long delays in access.

**What is added by this report?**

This report provides a basis for utilizing newspaper obituaries and funeral home reports to identify spikes in excess mortality more quickly.

**What are the implications for future research?**

The ability of newspaper obituaries and funeral home reports to evaluate “real time” changes in excess mortality should be evaluated in other regions of the country, including urban areas and other, non–Appalachian communities.

## Figures and Tables

Month (2020)	Funeral Home Listing Percent Difference	Obituary Percent Difference	TDH Washington County Percent Difference
January	−10.81%	−6.41%	0.75%
February	3.45%	19.32%	11.08%
March	−18.28%	13.77%	11.63%
April	−7.60%	20.71%	25.23%
May	33.54%	1.67%	6.22%
June	16.74%	38.87%	26.27%
July	16.88%	−2.84%	13.93%
August	−9.73%	−2.78%	−4.45%
September	14.00%	39.14%	29.73%
October	59.32%	34.21%	26.15%
November	20.75%	29.65%	37.29%
December	102.99%	70.63%	90.82%

**Table 1:** Calculated Excess Mortality and COVID–19 Attributed Excess Mortality in Washington County, TN, 2020.

Month	Expected Deaths	Actual (Reported) Deaths	Number of Excess Deaths	Percent Excess Deaths	COVID-19 Reported Deaths	Percent of Excess Deaths Attributed to COVID–19	Excess Deaths Not Directly Attributed to COVID–19	Percent of Excess Deaths Not Directly Attributed to COVID–19
**1st Quarter**								
January	134	135	1	0.75%	-	0.00%	1	100.00%
February	123	137	14	11.08%	-	0.00%	14	100.00%
March	129	144	15	11.63%	-	0.00%	15	100.00%
Total	386	416	30	7.68%	-	0.00%	30	100.00%
**2nd Quarter**								
April	107	134	27	25.23%	-	0.00%	27	100.00%
May	123	131	8	6.22%	-	0.00%	8	100.00%
June	118	149	31	26.27%	-	0.00%	31	100.00%
Total	348	414	66	18.85%	-	0.00%	66	100.00%
**3rd Quarter**								
July	122	139	17	13.93%	2	11.76%	15	88.24%
August	142	136	(6)	−4.22%	11	−183.33%	0	0.00%
September	123	160	37	29.73%	25	68.18%	12	31.82%
Total	388	435	47	12.21%	38	80.28%	9	19.72%
**4th Quarter**								
October	124	156	32	26.15%	21	64.95%	11	35.05%
November	138	189	51	37.29%	49	95.45%	2	4.55%
December	105	201	96	90.82%	57	59.58%	39	40.42%
Total	367	546	179	48.91%	127	70.82%	52	29.18%
**2020 Overall**								
Total	1,489	1,811	322	21.63%	165	51.24%	157	48.76%

NOTES:

* 2020 Expected Deaths were calculated using 2017–2019 TDH mortality data.

Actual Reported Deaths in 2020 were obtained through a TDH data request. Number of Excess Deaths is the difference between 2020 Expected and 2020 Actual Reported Deaths. Percent Excess Deaths was calculated by dividing the Number of Excess Deaths by 2020 Expected Deaths. COVID-19 Reported Deaths in 2020 were obtained through TDH Downloadable Datasets. Percent of Excess Deaths Attributed to COVID–19 was calculated by dividing COVID-19 Reported Deaths in 2020 by Number of Excess Deaths.

**Table 2:** Selected Funeral Home Listings in Washington County, TN, 2020*

Month	2020 Expected Reports	Actual Funeral Home Reports 2020	Number of Excess Funeral Reports	Percent Excess Funeral Reports
**1st Quarter**				
January	99	88	(11)	−10.81%
February	77	80	3	3.45%
March	93	76	(17)	−18.28%
Total	269	244	(25)	−9.29%
**2nd Quarter**				
April	83	77	(6)	−7.60%
May	55	73	18	33.54%
June	74	86	12	16.74%
Total	212	236	24	11.50%
**3rd Quarter**				
July	77	90	13	16.88%
August	75	68	(7)	−9.73%
September	83	95	12	14.00%
Total	236	253	17	7.36%
**4th Quarter**				
October	59	94	35	59.32%
November	80	97	17	20.75%
December	56	113	57	102.99%
Total	195	304	109	55.90%
**2020 Overall**				
Total 2020	911	1,037	126	13.79%

NOTES:

* 2020 Expected Reports were calculated using 2017–2019 funeral home data to create projections.

Actual Funeral Home Reports 2020 were obtained from funeral home websites. Number of Excess Funeral Reports is the difference between 2020 Expected and 2020 Actual Reported Deaths. Percent Excess Funeral Reports was calculated by dividing the Number of Excess Funeral Reports by 2020 Expected Reports.

**Table 3:** Selected Obituary Reports in Washington County, TN, 2020*

Month	Expected Reports	Actual Newspaper Obituaries	Number of Excess Newspaper Obituaries	Percent Excess Newspaper Obituaries
**1st Quarter**				
January	154.67	146	(8.67)	−5.60%
February	117.33	140	22.67	19.32%
March	138.00	157	19.00	13.77%
Total	410.00	443	33.00	8.05%
**2nd Quarter**				
April	112.67	136	23.33	20.71%
May	120.00	122	2.00	1.67%
June	112.33	156	43.67	38.87%
Total	345.00	414	69.00	20.00%
**3rd Quarter**				
July	141.00	137	(4.00)	−2.84%
August	144.00	140	(4.00)	−2.78%
September	124.33	173	48.67	39.14%
Total	409.33	450	40.67	9.93%
**4th Quarter**				
October	126.67	170	43.33	34.21%
November	132.67	172	39.33	29.65%
December	126.00	215	89.00	70.63%
Total	385.33	557	171.67	44.55%
**2020 Overall**				
Total 2020	1,550	1,864	314	20.28%

NOTES:

* 2020 Expected Reports were calculated using 2017–2019 funeral home data to create projections.

Actual Funeral Home Reports 2020 were obtained from funeral home websites. Number of Excess Funeral Reports is the difference between 2020 Expected and 2020 Actual Reported Deaths. Percent Excess Funeral Reports was calculated by dividing the Number of Excess Funeral Reports by 2020 Expected Reports.
